# Research progress of stimulus-responsive antibacterial materials for bone infection

**DOI:** 10.3389/fbioe.2022.1069932

**Published:** 2022-12-23

**Authors:** Changqing Wang, Peng Xu, Xiaoxu Li, Yuhao Zheng, Zhiming Song

**Affiliations:** Department of Sports Medicine, Orthopaedic Center, The First Hospital of Jilin University, Changchun, China

**Keywords:** stimulus response, infection, antibiotics, nanomaterials, metal nanoparticles, antimicrobial peptides, drug resistance

## Abstract

Infection is one of the most serious complications harmful to human health, which brings a huge burden to human health. Bone infection is one of the most common and serious complications of fracture and orthopaedic surgery. Antibacterial treatment is the premise of bone defect healing. Among all the antibacterial strategies, irritant antibacterial materials have unique advantages and the ability of targeted therapy. In this review, we focus on the research progress of irritating materials, the development of antibacterial materials and their advantages and disadvantages potential applications in bone infection.

## 1 Introduction

Bacterial infection was an important challenge in the fields of medicine, environment or food, etc. Antibiotics play an indelible role as an important drug to control infection. However, the resistance of bacteria to antibiotics is gradually increasing, due to the previous neglect of drug management (Rasigade and Vandenesch, 2014; Tacconelli et al., 2018). Methicillin-resistant *Staphylococcus aureus*, vancomycin intermediate *Staphylococcus aureus*, vancomycin-resistant enterococci and *Clostridium* labile are common species with antibiotic resistance (AMR) or multiple drug resistance (MDR). According to the statistics from World Health Organization (WHO), lower respiratory tract infection was the fourth leading cause of death, from 2000 to 2019. AMR is one of the greatest dangers to global health and development. Bacterial infection can lead to sepsis, bacteremia and even death. By 2050, 10 million people are expected to die from diseases caused by bacteria and other microbial infections (da Rosa et al., 2020).

Compared with other infections, bone tissue infections are more difficult to diagnose and treat, especially those involving bone related implants (Tande Aaron and Patel, 2014). The main infection routes of osteomyelitis, purulent arthritis and implant related infection are blood borne, adjacent tissue infection, or infection after trauma, surgery or foreign body implantation (such as joint replacement) (Brady et al., 2006; Wright and Nair, 2010). In orthopedic implant infection, *staphylococcus* infection accounts for two-thirds of all pathogen infections. Implant infection is prone to repeated infection, leading to chronic bone infection. It is unlikely to rely solely on antibiotic treatment, which is more difficult to cure than other infections. Many bone infections still require antibiotic treatment for 4–6 weeks after surgical debridement (Marculescu et al., 2006; Del Pozo and Patel, 2009; Lee et al., 2010). Bone loss is a major complication of osteomyelitis. The osteoclast precursor of bone marrow is induced to differentiate into active macrophages by live *Staphylococcus aureus*, and secretes many proinflammatory cytokines. These cytokines can enhance the bone absorption capacity of mature osteoclasts and promote the differentiation of uninfected osteoclasts (Trouillet-Assant et al., 2015).

Bone infection can be caused by continuous transmission of surrounding tissues, direct bone trauma caused by surgery or injury, or blood borne transmission caused by systemic bacteremia. It is still a major medical burden. In the United States, there are about 22 cases per 100,000 people, and the incidence rate has been rising, especially among the elderly and diabetes patients. The infection of bone is mainly due to the destruction of the Haves system, the loss of blood supply support and necrosis of bone, the inability to remove bacteria, the formation of pus cavity or bone erosion, resulting in prolonged healing and repeated attacks. Soft tissue infection mainly occurs in areas with sufficient blood supply. The pus can be drained out through surgery or the granulation tissue can be promoted by changing the dressing. The granulation tissue with blood supply can be cured when it grows and heals.

At present, there are two main strategies to deal with infection: One is to build an antifouling surface to resist the adhesion of bacteria, and the other is to use fungicides to kill bacteria (Campoccia et al., 2013; Berne et al., 2018). Bacteria can produce extracellular matrix on the contact surface to form a biofilm to protect themselves, which usually requires 10 to 1,000 times the concentration of antibiotics to completely remove the bacteria (Yang et al., 2012; Wang et al., 2016). Bacteria that produce biofilms are resistant to antibiotics, which explains the root cause why biofilm removal is difficult. The uptake of nutrients by the outer layer of the biofilm is faster than that of the inner layer, and it has higher metabolic activity and faster growth rate, which makes it more difficult to develop effective antibiotics. The biofilm outer layer cells take in nutrients faster than the inner layer cells, and have higher metabolic activity and faster growth rate, which makes it more difficult to develop effective antimicrobials (Werner et al., 2004; Stewart and Franklin, 2008).

In the 1970s, the stimulus response system was first introduced, beginning with the use of locally released drugs from thermosensitive liposomes (Wang et al., 2018). In recent years, due to the sensitivity to external environmental signals or pathological abnormalities, the release rate of antimicrobials was adjusted when needed to effectively kill bacteria. Irritant response materials have made great progress in the field of antimicrobials (Lu et al., 2016; Wei et al., 2017). This review mainly introduces the source and research progress of irritating materials, the development of antibacterial materials and their advantages and disadvantages.

## 2 Stimulus-responsive antibacterial material

In the last century, people began to make and utilize stimulus-responsive systems. In the seventies, it has been reported that by heating neomycin-containing liposomes to their phase transition temperature to control the release of antibacterial drugs, inhibit the protein synthesis of *E. coli* and kill bacteria (Kim et al., 2017). Thermally responsive stimulation systems have evolved in subsequent studies and mainly include polymer-solvent mixtures transitioning from single-phase systems at low temperatures to two-phase systems at high temperatures and polymer-solvent mixtures transitioning from two-phase systems at low temperatures to single-phase at high temperatures (Schmaljohann, 2006; Zhao et al., 2019). In the early 20th century, von Tappeiner described the principles that underlie photodynamic therapy (PDT) (Felsher, 2003; Li et al., 2018a). Due to the lack of an ideal molecular photosensitizer and an efficient activation process, the full ideal of PDT has not yet been realized (Lovell et al., 2010). Through so many years of development, many nanodelivery systems have been designed to overcome these problems (Lucky et al., 2015; Li et al., 2018a). PDT is highly efficient, spatiotemporal selective, not easy to produce drug resistance, and its killing effect is limited to tens of nanometers. However, most current PDT systems promote the formation of ROS through type II mechanisms, which makes the activation of PDT systems very dependent on oxygen (Mukai et al., 2018). In recent years, some scholars have also studied the use of type I mechanism photosensitizers to reduce dependence on oxygen (Huynh and Zheng, 2013; Li et al., 2018b). Due to the composition of multiple components, especially materials with low biocompatibility, most PDT systems require cumbersome toxicity studies and complex manufacturing procedures, and clinical application is still a difficult challenge. Magnetic drug-controlled delivery was first proposed in the 80s of the last centuries (Williams et al., 2009; Price et al., 2018). In later developments, more versatile magnetic probes were developed that allowed for a combination of diagnosis and treatment, including targeted drug delivery and drug delivery, among others (Peng et al., 2017; Tang et al., 2018). The magnetic field is largely unabsorbed by tissue, penetrates deeper than infrared and visible light, and philately is easy to use and the ability to target carriers deeper into tissues (Kumar and Mohammad, 2011; Owen et al., 2012). External magnetic fields can increase heat in magnetic nanoparticles for local hyperthermia, or increase polymer permeability to the matrix or disrupt temperature-sensitive drug envelopes to induce drug release (Kumar and Mohammad, 2011; Thirunavukkarasu et al., 2018). However, using an external magnet to attract a magnetic drug carrier is difficult to accurately locate to an area below 5 cm under the skin, and there is a lack of mechanism for delivery to the depths of the body (Shapiro, 2009; Price et al., 2018). In the 90s, pH response systems have reported that when ionizable groups are attached to polymers, they cause conformational changes in soluble polymers as well as changes in hydrogel swelling (Nakamae et al., 1992; Miyata et al., 1994; Nakamae et al., 1997). pH differences between tissues have been widely used in the design of pH-sensitive drug delivery systems in stimuli-responsive drug delivery systems for their thermal/chemical stability, polymer species morphology, and biocompatibility (Nakamae et al., 1992; Zhu and Chen, 2015; Shin et al., 2021). At the beginning of the 21st century, polymers with enzyme-responsive systems have been considerably developed in the field of hydrogels and nanoparticles (Ulijn, 2006; Ghadiali and Stevens, 2008; Hahn and Gianneschi, 2011). As a key component of bionanotechnology, enzymes have excellent biorecognition capabilities and excellent catalytic performance, and the combination of enzymes and nanomaterials has been successfully used in diagnosis and drug treatment (Andresen et al., 2005; Minelli et al., 2010). The precise identification of substrates by enzymes can effectively reduce the amount of drugs used and thus reduce the toxicological effects of drugs without ensuring efficacy (Minelli et al., 2010). Over the past 20 years, great progress has been made in the development of redox-responsive nanocarriers, typically glutathione-responsive nanocarriers (Cheng et al., 2011; Lee et al., 2013). As an ideal internal stimulator, glutathione can be used for the rapid unstable resolution of intracellular nanocarriers, and this method of drug delivery acting on intracellular cells is beneficial to overcome multidrug resistance and reduce drug side effects (Li et al., 2020a). In the 90s, there was an interest in precisely controlled drug delivery by electrical stimulation (Pernaut and Reynolds, 2000; Murdan, 2003; Abidian et al., 2006). Compared to other stimuli, electrical stimulation can be applied to biological systems quickly, reversibly and locally. Thanks to the development of MEMS, implantable devices can shine in controlled drug release (Schmidt et al., 2010). Stimulus-responsive materials are divided into endogenous and exogenous response systems. According to the type of response factors, the endogenous stimulus response system is divided into PH responsiveness, redox sensitivity, enzyme responsiveness, etc. Exogenous stimulus response system includes light sensitivity, ultrasonic trigger, magnetic trigger, electrical trigger, etc. Multiple stimulus response systems have developed rapidly in recent years, such as: topologically integrating temperature-responsive Nisopropylacrylamide (NIPAM), photoresponsive azobenzene/cyclodextrin (Azo/CD) complex, hydrophilic PHEMA segments, and nanobactericides (AgNPs) on one single substrate (Ni et al., 2021). The antibacterial substances in the stimulus response system are constantly updated and optimized. Now the mainstream antibacterial substances are antibiotics, metal nanomaterials, cationic antimicrobial agents and antimicrobial enzymes, each of which has its own advantages and disadvantages. Stimulus-responsive materials have good development and application prospects in the field of medicine, such as medical equipment, drug delivery, therapeutic diagnostics, tissue engineering, etc. (Smith et al., 2012; Duque Sanchez et al., 2016; Gao et al., 2017; Morgese et al., 2018; Zhou et al., 2018; Xue et al., 2019) The stimulus response system is shown in Table 1.

**TABLE 1:** Stimulus response materials.

Response type (material)	Loading drugs	Loading pathway	Biological evaluation	Reference
Glutamyl endonuclease	AgNPs encapsulated by mesoporous silica nanoparticles (MSN) (MSN-Ag)	Physical encapsulation	***In vitro.*** (*Staphylococcus aureus*) and *in vivo* (rat)	Ding et al. (2020)
serine protease-like B enzyme proteins (SplB)	AgNO_3_	Physical encapsulation	***In vitro.*** (MRSA) and *in vivo* (rat MRSA infection model)	Zuo et al. (2020)
Lipase	Vancomycin	Physical encapsulation	***In vitro.*** (*Staphylococcus aureus*)	Xiong et al. (2012)
β-lactamase	Nanoparticles (AgNPs, etc.)	Physical encapsulation	***In vitro.*** (*Staphylococcus aureus*, *Klebsiella pneumoniae*, *Pseudomonas aeruginosa*, cloaca and *Bacillus cereus*)	Alkekhia et al. (2022)
polyDVBAPS	TCS (triclosan)	Drug binding	***In vitro.*** (*Escherichia coli* and *Staphylococcus aureus*)	Wang et al. (2019)
polyDVBAPS	AgNPs	Physical encapsulation	***In vitro.*** (*Escherichia coli* and *Staphylococcus aureus*)	Zhang et al. (2018)
Salt responsiveness	Poly (trimethylamino) ethyl methacrylate (pTMAEMA)	Drug binding	***In vitro.*** (*Staphylococcus epidermidis Escherichia coli*)	Huang et al. (2019)
human cathelicidin LL-37	Antibacterial peptides (AMP)	Physical encapsulation	***In vitro.*** (*Escherichia coli*)	Gontsarik et al. (2019)
pH-sensitive quaternary pyridinium salt (QPS)	(E)-1-hexadecyl-4-((4-(methacryloyloxy)phenyl)diazenyl)-pyridinium bromide (named Azo-QPS-C16)	Drug binding	***In vitro.*** (*Escherichia coli* and *Mutant streptococci*)	Yang et al. (2018)
poly (N-vinylpyrrolidone-co-N-vinylformamide)	Doxorubicin	Drug binding	***In vitro***	Peng et al. (2019)
Light stimulus response	Reactive oxygen species produced by excitation (ROS)	Drug binding or physical encapsulation	***In vitro.*** (*Escherichia coli*)	Chong et al. (2012)
phthalocyanine molecules (NanoPcA)	Reactive oxygen species produced by excitation (ROS)	Drug binding	***In vitro.*** (*Escherichia coli* and *Staphylococcus aureus*)	Li et al. (2018b)
P(BMA-co-AAm-co-MAA)	AgNPs and ofloxacin	Physical encapsulation	***In vitro***	Amoli-Diva et al. (2017)
PEDOT		Drug binding	***In vitro.*** (*Salmonella typhimurium*)	Gomez-Carretero et al. (2017)
Magnetic stimulus response	Isoniazid (INH)	Physical encapsulation	***In vitro***	Zhao et al. (2017)
Fe3O4 MNP	Vancomycin	Physical encapsulation	***In vitro***	Harris et al. (2017)

Poly DVBAPS: poly (3-(dimethyl (4-vinylbenzyl) ammonio) propyl sulfonate). P (BMA-co-AAm-co-MAA): poly butyl methacrylate-co-acrylamide-co-methacrylic acid. PEDOT: poly (3,4-ethylenedioxythiophene). MNP: superparamagnetic nanoparticles.

### 2.1 Endogenous stimulus response system

#### 2.1.1 Enzyme responsive material

**FIGURE 1:**
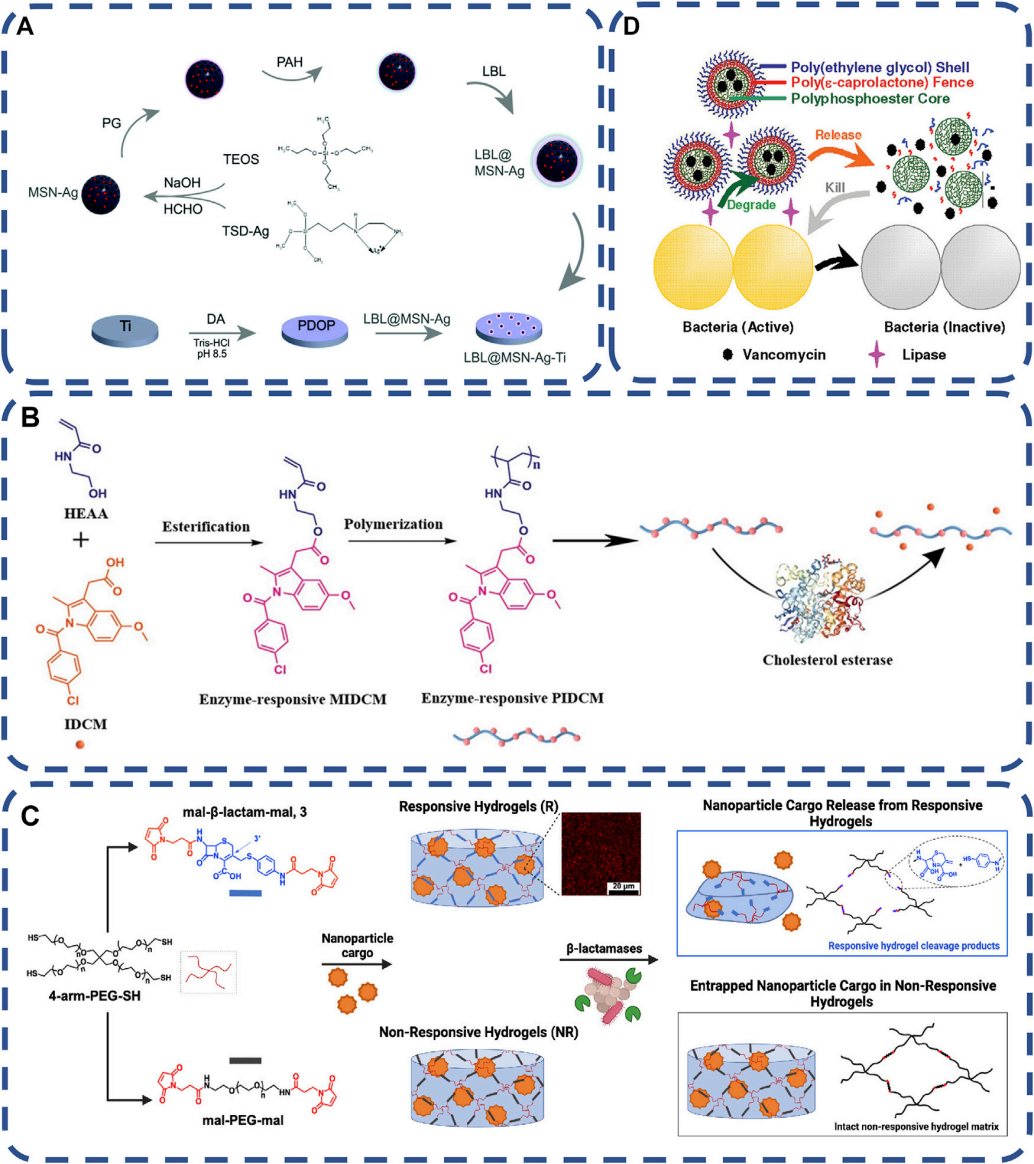
**(A)** schematic diagram of preparation process of LBL@MSN-Ag modified Ti substrate (Ding et al., 2020). Copyright 2020, Biomater Sci. **(B)** Schematic diagram of on-demand drug delivery triggered by bacterial lipase in the treatment of bacterial infection using polymerized three-layer nanogels (TLN) (Xiong et al., 2012) Copyright 2012, J Am Chem Soc. **(C)** Schematic Representation of β-Lactamase-Responsive (R) Hydrogel Fabrication *via* Thiol–ene Michael-Type Addition, β- The hydrolysis of lactam leads to the splitting of the main chain of the polymer, which releases nanomaterials, and the manufacturing process and stability of unresponsive hydrogels (Alkekhia et al., 2022) Copyright 2022, ACS Appl Mater Interfaces. **(D)** Schematic showing the synthesis procedures of the enzyme-responsive prodrug that allows for the triggered release of indomethacin (Ye et al., 2020). Copyright 2020, Macromol Biosci.

In the past 10 years, enzyme responsive polymers have made great progress, Many enzymes have been used in the design of antimicrobial stimuli responsive drugs (Ma et al., 2020a), such as, lipase, phosphatase, protease, etc. As the most important catalytic substance in the body, enzymes play an important role in a series of physiological processes, and their expression levels will also change in the state of disease. The high selectivity and biocompatibility of enzyme responsive materials determine that they can play a role in many biomedical applications. As the original trigger factor, the change of specific enzyme expression level can cause enzyme responsive biomaterial reaction and control the release of antibacterial drugs at the required sites, so as to kill bacteria。Enzymes are highly expressed in the infected site, and some specific enzymes were used to identify stimuli, thus achieving the release of antibacterial substances (Wei et al., 2022). Glutamyl endonuclease (V8 enzyme) is a product of *Staphylococcus aureus*. Ag nanoparticles were encapsulated in mesoporous silica nanoparticles (MSN), and then poly L-glutamic acid (PG) and polyallylamine hydrochloride (PAH) were assembled layer by layer on MSN-Ag to form LBL@MSN-Ag nanoparticles. When V8 enzyme cleaves the PG amide bond, the Ag ion in the polymer is released, thus killing bacteria at the bacterial infection site (Ding et al., 2020), as shown in Figure 1A. Through the reaction with the substrate, the enzyme achieves the precise location of bacteria and control the release of antimicrobials, thus killing bacteria (Hu et al., 2012; Haas et al., 2015; Zuo et al., 2020). It is also a good strategy to kill drug-resistant bacteria. In addition to specific enzymes, wound infection causes macrophages to accumulate and secrete cholesterol esterase cholesterol esterase (CE) in the wound, which catalyze the hydrolysis of ester bonds to sterols and fatty acids, and be used to prepare enzyme-responsive antibacterial materials (Ye et al., 2020), as shown in Figure 1B.

Most *Staphylococcus aureus*, some *Escherichia coli* and *Pseudomonas aeruginosa* can produce lipase and connect ciprofloxacin to the surface of polyethylene glycol (PEG) by anhydride, which shows good biocompatibility in physiological conditions. Once a bacterial infection occurs, the bacteria begin to secrete lipase, and the polymer releases ciprofloxacin, which almost completely kills the bacterial strain at the initial stage of biofilm formation (Hasan et al., 2009; Xiong et al., 2012), as shown in Figure 1C.

The production of β-lactam hydrolase by bacteria is the main cause of bacterial drug resistance, which could be produced by a variety of bacterial pathogens. Maleimide functionalized cephalosporins were used as cross-linking agents by terminal cross-linking polymerization with polyethylene glycol macromonomers, to development of hydrogels for specific degradation of β-lactamases as a platform to trigger drug delivery, which could effective against infection without increasing the dose (Alkekhia et al., 2022), as shown in Figure 1D. Enzyme stimulation response system can overcome the challenges of bacterial drug resistance, controlled release of antibiotics and biofilm formation. It has high histocompatibility and great potential in the field of biomedicine.

#### 2.1.2 Salt responsive material

At the earliest time, some scholars studied the ability of zwitterionic polymers to repeatedly switch from bactericidal cationic form to protein repellent zwitterionic form under controlled switching in alkaline/acidic solutions (Cao et al., 2012). Later, more and more researchers began to pay attention to the development of materials based on this strategy. In recent years, the research on the sterilization and release of salt responsive materials has made progress. The regenerative surface of polyampholytic ions shows a transition between the repulsive state of biomolecules and the adhesion state of biomolecules when the counter ion type and the concentration of responsive salts are used (Li et al., 2020b). It is the most commonly used method to achieve sterilization and release functions by introducing bactericides to achieve the regeneration of antibacterial surfaces.

**FIGURE 2:**
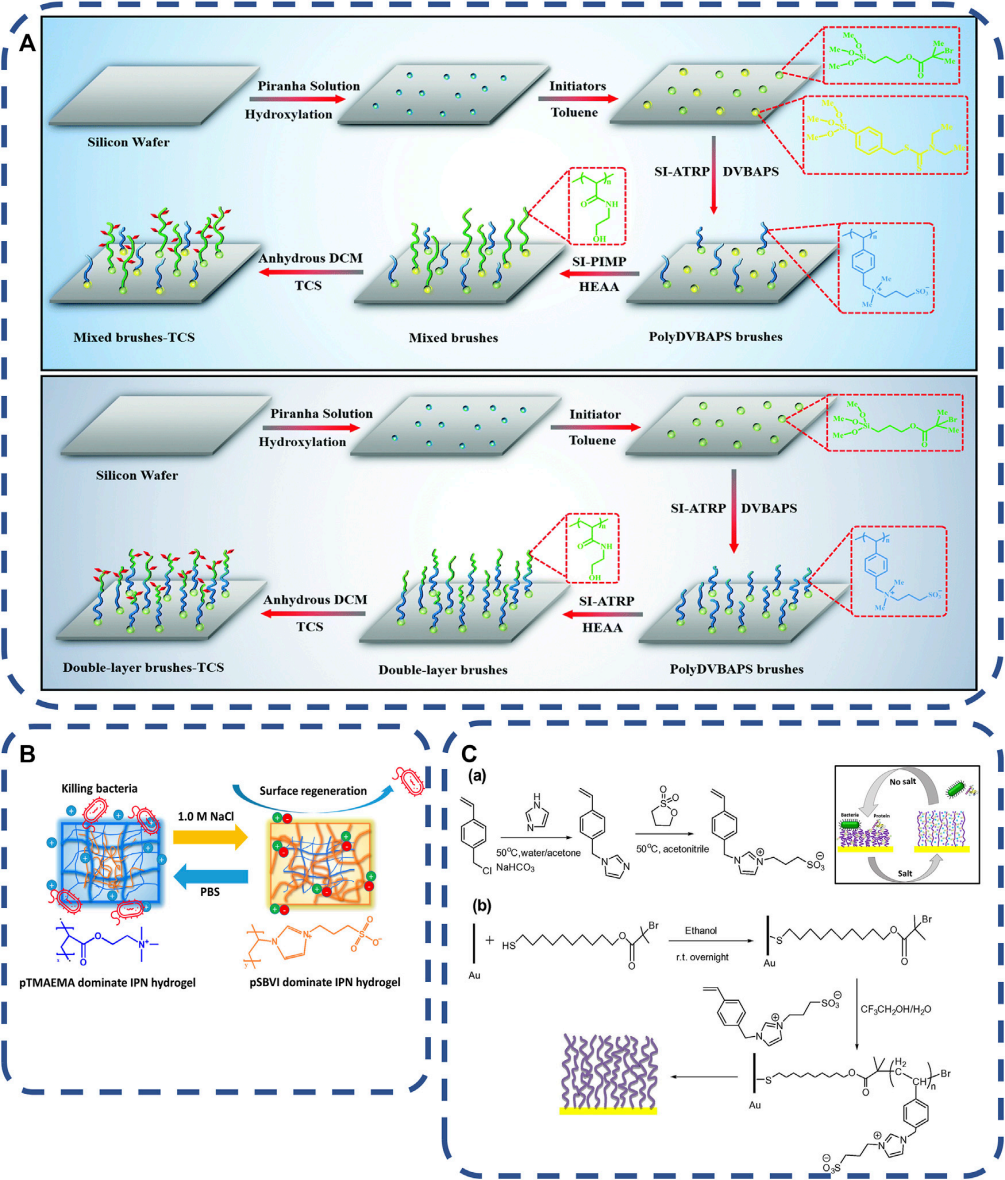
**(A)** Schematic of the two types of antibacterial polymer brushes with hierarchical structures of a mixed polyDVBAPS/polyHEAA brush and a double-layer poly (DVBAPS-b-HEAA) brush (Wang et al., 2019). Copyright 2019, J Mater Chem B. **(B)** schematic diagram of exchangeable salt to kill bacteria and IPN hydrogel to release bacteria in PBS and NaCl solutions (Huang et al., 2019). Copyright 2019, Biomacromolecules. **(C)** (a) Synthesis of VBIPS monomer in response to salt and (b) schematic diagram of SI-ATRP process by brushing the poly vein double shrinkage analyzer to the gold surface coated with fixed initiator (Chen et al., 2016). Copyright 2016, Acta Biomater.

Mainly, Salt-responsive materials are used to eliminate bacteria-release dead bacteria to prevent bacterial attachment and biofilm formation (Sundaram et al., 2014; Wang et al., 2019). In the process of contact killing strategy, the accumulation and covering of dead bacteria on the antibacterial surface leads to the decrease of antibacterial effect, that develop new materials with dual characteristics of antifouling and antibacterial are necessarily (Blummel et al., 2007; Klein et al., 2011; Ma et al., 2016). Some studies have integrated salt-responsive PolyDVBAPS (poly (3-(dimethyl (4-vinylbenzyl) ammonium) propyl sulfonate), antifouling PolyHEAA (poly (N-hydroxyethyl acrylamide)) and bactericidal TCS (triclosan) onto a single surface. PolyDVBAPS and PolyHEAA were polymerized and grafted onto the substrate in different ways to form two kinds of PlyDVBAPS/Poly (HEAA-G-TCS) with different hierarchical structures, As shown in Figure 2A. The polymer has three functions of preventing bacterial attachment, killing attached bacteria on the germicidal surface and stimulating the response material to release bacteria from the surface, and the salt response material surface has high regeneration ability (Wang et al., 2019). Salt-responsive materials are usually anti-fouling, sterilization, release of three different properties of materials together, have the ability of regeneration and in a short time can play a role for many times (Sundaram et al., 2014; Zhang et al., 2018). However, it is easily affected by the environment, such as electrolyte concentration.

Salt responsive renewable surface is an ideal choice for antibacterial materials. Huang et al. (2019) synthesized salt responsive pTMAEMA/pSBVI hydrogels using antibacterial hydrogel poly (trimethylamino) ethyl methacrylate (pTMAEMA) and antifouling material amphoteric poly (sulfonbutylvinylimidazole) (pSBVI), As shown in Figure 2B. The bactericidal rate and release rate did not show obvious reusability after five germicidal release cycles. As shown in Figure 2C. Chen et al. (2016) issued salt-responsive polymer brushes of poly (3-(1-(4vinylbenzyl)-1H-imidazol-3-ium-3-yl) propane-1-sulfonate) (polyVBIPS) to realize the reversible and repeated switching of protein capture/release and surface wettability in a controllable square. The reversible switching of multiple cycles is shown in the table from ∼ 40°to 25°. Here, the collapsed chain conformation is used to achieve surface adhesion at low ionic strength, and the extended chain conformation is used to achieve antifouling performance at high ionic strength.

#### 2.1.3 PH responsive materials

**FIGURE 3:**
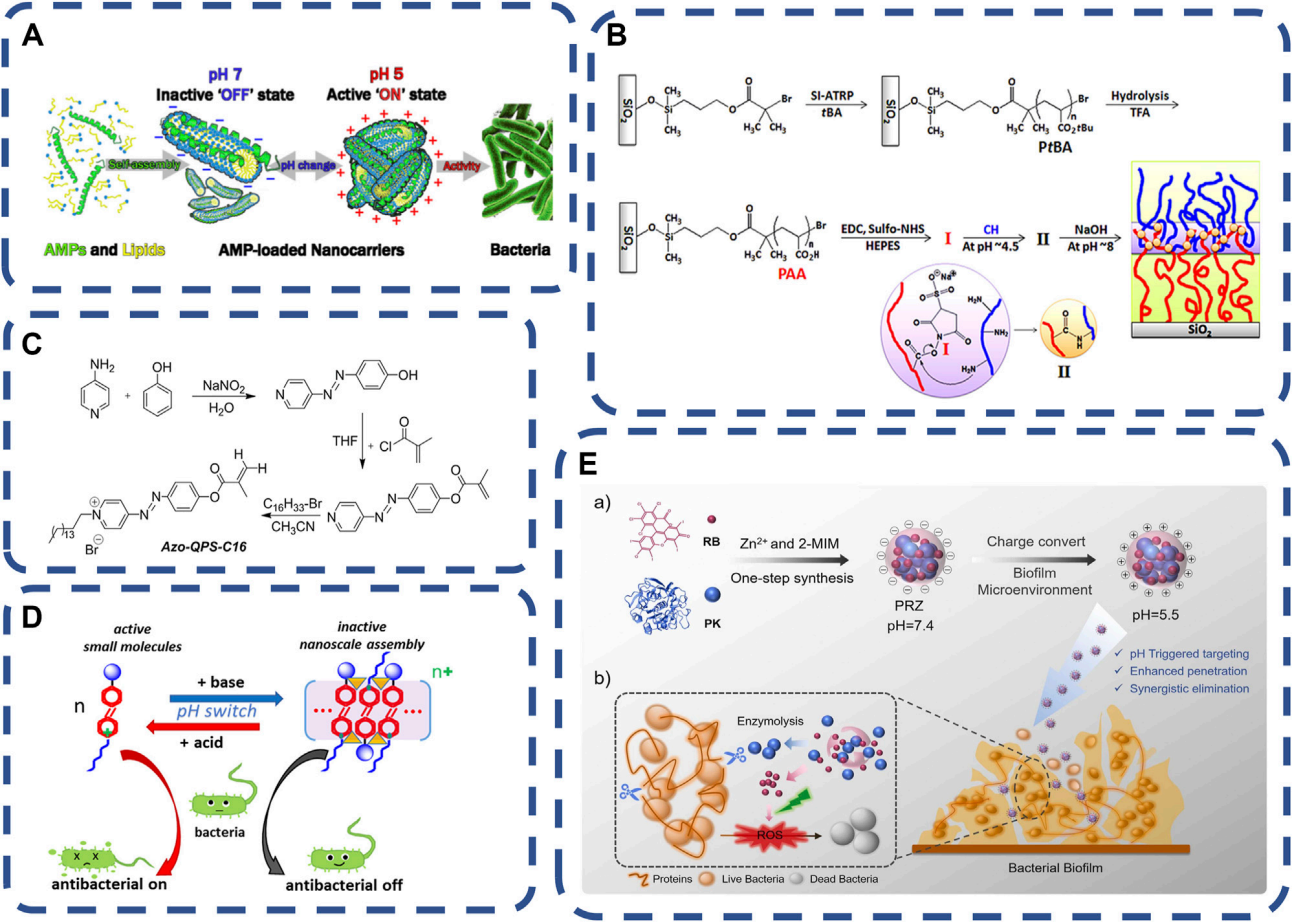
**(A)** Amphiphilic antimicrobial peptides are transformed from core-shell cylindrical micelles with a cross-sectional diameter of 5.5 nm and a length of 23 nm at pH 7.0 to branched chain linear micelle aggregates at pH 5.0. At pH 5.0, positively charged LL-37/OA aggregates have high antibacterial activity (Gontsarik et al., 2019). Copyright 2019, ACS Appl Mater Interfaces. **(B)** Synthesis of pH responsive materials (Lee et al., 2015).Copyright 2015, Biomacromolecules. **(C,D)** pH responds to the characteristics and assembly behavior of azo QPS-C16 (Yang et al., 2018). Copyright 2018, ACS Appl Mater Interfaces. **(E)** Synthesis and mechanism of pH responsive PRZ (Ding et al., 2022) Copyright 2022, ELSEVIER.

The pH of blood is maintained by the dynamic stability of the concentration of carbon dioxide, bicarbonate and other examples. Under some pathological conditions, tissues will have pH values different from the physiological pH values. The local acidic environment of the infected site is caused by immune reaction and anaerobic glycolysis (Ma et al., 2020b). This feature makes researchers begin to develop pH responsive carriers. pH responsive carriers have been developed earlier in the field of tumor therapy, similar to tumor microenvironment (Kanamala et al., 2016). The microenvironment of the infection site is different from that of normal tissues. Therefore, pH responsive nano carriers also have a greater impact in the field of bacterial infection therapy. The change of pH affects the state of the gel (the transition between gel and solution), significantly. pH response materials can respond to bacterial acids, such as acetic acid, lactic acid and malic acid, etc. In PH response system, polyacrylamide (PAAm) (Lee et al., 2015; Bellingeri et al., 2018; Zhong et al., 2018), tannic acid (TA) (Jing et al., 2019), polyacrylic acid (PAA) (Modarresi-Saryazdi et al., 2018; Raju et al., 2018), polymethacrylic acid (PMAA) (Rasib et al., 2018; Sharpe et al., 2018; Qu et al., 2019) and chitosan (CS) (Zhou et al., 2013) are widely used because of their biocompatibility. Cross-linking agents such as glutaraldehyde, carbodiimide, transglutaminase, Glyoxal and binders with bifunctional groups are commonly used in the chemical modification of hydrogels (Kaul and Amiji, 2005; Lee et al., 2012). They could bind ammonium ions produced by infected bacteria and release antibacterial materials such as antimicrobial peptides (AMP) (Traba and Liang, 2015). Local pH environmental changes trigger structural changes, locally activate the bactericidal effect of antibacterial substances, and improve germicidal efficacy (Gontsarik et al., 2019), as shown in Figure 3A. Yang et al. (2018) reported a new pH-sensitive quaternary pyridine salt (QPS). Its antibacterial activity is enhanced when the low pH value decreases, and the activity can be controlled by regulating pH between four and eight of pH. The compound selectively inhibits the growth of acid-producing bacteria, which could be used to kill acid-producing bacteria and to regulate the pH value of the environment to provide antibacterial protection, as shown in Figure 3B pH responsive polymers were designed to be responsive to a specific range of environmental pH, controlling drug release and killing bacteria only in the target tissue (Du et al., 2010; Liechty et al., 2013; Sim et al., 2017).

In clinical application, pH responds to the direct contact between the outer layer of the germicidal system and the tissue, requiring the outer layer material pH responsiveness, biocompatibility, inner layer response to stimulation and drug adjustable release properties (Lee et al., 2015; Gontsarik et al., 2019). Such as a charge-switchable and pH-responsive nanocomplex is fabricated *via* a facile aqueous one-pot zeolitic imidazolate framework-8 (ZIF8) encapsulation of proteinase K(PK) and photosensitizer Rose Bengal (RB), for enzymatic and photodynamic therapies (PDT) against biofilm infections (Ding et al., 2022). The nanocomposite (PRZ) is negatively charged in physiological environment and becomes positively charged when stimulated by acidic substances. Positive charge can enhance the penetration of PRZ into the biofilm and promote competition and the release of RB. RB produces reactive oxygen species under light to further eliminate the remaining bacteria. pH responsive materials were used for gastrointestinal infections, wound healing, treatment of osteomyelitis and implantable medical devices (Chung et al., 2014; Lee et al., 2015; Anandhakumar et al., 2016; Yang et al., 2018; Ding et al., 2022), as shown in Figure 3C.

#### 2.1.4 Redox response material

**FIGURE 4:**
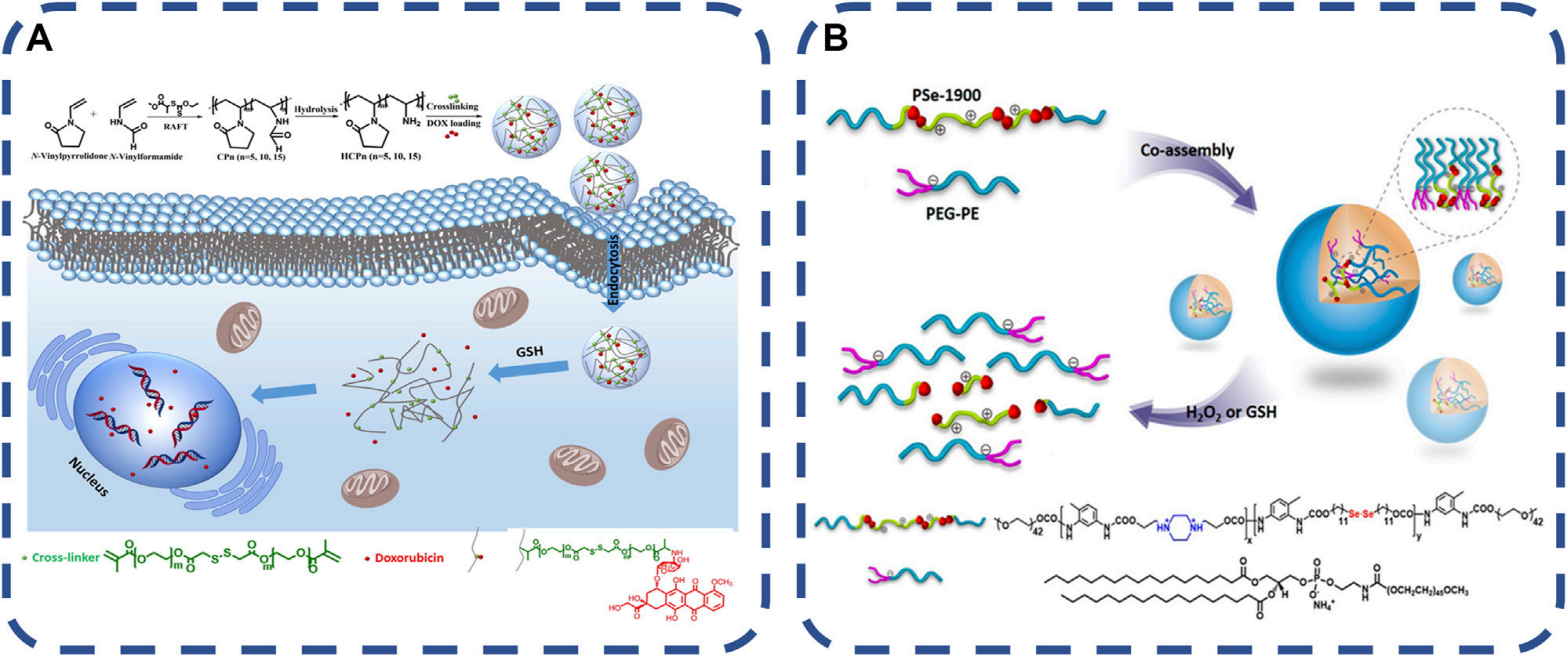
**(A)** redox response. The synthesis of prodrug nanogels is based on amine-functionalized polymers, which are integrated into fine cells in a reduced environment and trigger intracellular drug release (Peng et al., 2019).Copyright 2019, J Colloid Interface Sci. **(B)** Redox responsive co assemblies formed from block copolymers containing selenides and polymer lipids (Wang et al., 2014). Copyright 2014, Langmuir.

Bacterial infection produces metabolites, such as cysteine, glutathione, etc. In the past 10 years, the development of redox responsive materials has made great progress. Many redox responsive matrices have been developed to extend the cycle time and immediately release drugs at biologically relevant concentrations. Redox responsive nanocarriers can be designed from organic and/or inorganic materials. Inorganic nanomaterials have attracted more attention due to their unique physical and chemical properties and ability to experience the modification process. Liposomes, dendrimers, micelles or protein-based nanomaterials can be used as redox responsive nanocarriers for drug delivery. Some nanocarriers have been approved by the US Food and Drug Administration (FDA) and used in clinical practice. Inflammation lead to a high concentration of reactive oxygen species (ROS) at the infection site. The method of using redox responsive cross-linking agents with various functional groups to manufacture drug delivery carriers through a variety of synthesis strategies has made good progress (Howard et al., 2008). The basic principle is that the redox active substances embedded in the polymerization system will be cleaved by substances such as glutathione (GSH) when they are internalized by cells (Cao et al., 2014; Krisch et al., 2016). Pegylated nanogels are decorated by capturing, adsorbing or covalently grafting PEG chains. Cross-linking agents are the main components in the preparation of redox responsive nanogels, which are generally redox active units containing tellurium bonds, disulfide bonds and diselenide bonds, which are broken in response to redox triggers to achieve the effective release and degradation of antibiotics (Ren et al., 2012; Wang et al., 2014; Sun et al., 2017). The synthesis strategies of the polymer (Kumar et al., 2019) are as follows: 1. Free radical polymerization with redox responsive cross-linking agent; 2. Adding redox response cross-linking agent to ring-opening polymerization; 3. Michael Additionreaction method, coupling a large number of nucleophiles with electron-deficient olefins to synthesize a simple and efficient dynamic controlled polymerization of hyperbranched polymers under mild conditions. 4. Self-crosslinking of mercaptan groups. Two polymer chains containing mercaptan (SH) side chains are cross-linked by a simple thiol-disulfide bond exchange reaction to form nanogels. 5. Disulfide crosslinked branched chain nanogels have the potential to be used as redox responsive drug delivery carriers. Peng et al. (2019) synthesized a series of water-soluble poly (N-vinylpyrrolidone-co-N-vinylformamide) copolymers. The copolymers were hydrolyzed under alkaline conditions to obtain primary amine functional reactive copolymers. After that, the copolymer and doxorubicin (DOX) were covalently coupled with the redox response crosslinking agent in the water-in-oil emulsion, and the prodrug nanogels were formed by Michael addition, as shown in Figures 4A, B. The gel has good biocompatibility and sustained drug release. Advanced technologies related to nanosynthesis can provide better drug delivery carriers, which can show their role in the precise location of lesions, and continue to develop towards highly sensitive materials.

### 2.2 Exogenous stimulus response system

#### 2.2.1 Light/heat triggered material

Photodynamic therapy (PDTA) began to be used for tumor treatment in the last century. In recent years, the drug resistance of traditional antibiotics has become increasingly serious. Due to the advantages of non-invasive and broad antibacterial properties, researchers have paid attention to it and designed a variety of light response systems to achieve on-demand drug release in response to light in specific wavelength areas. Moreover, PDTA has also demonstrated its strong ability to eliminate biofilm, and has now developed into a broad antibacterial system (Misba et al., 2018). Polymer antibacterial materials, light response materials induce antibacterial substances generally have two effective ways: photothermal triggering and photodynamic triggering. Compared with other response materials, the corresponding operation of light is simple, and the irradiation site, time and dose can be controlled, easily (Gohy and Zhao, 2013). Photoresponsive materials can be obtained by introducing photoresponsive groups, but most of the photoresponsive groups are sensitive to ultraviolet rays, such as azobenzene, o-nitrobenzyl ester and so on. Due to the low tissue penetration and phototoxicity of ultraviolet light, it is not suitable for practical application. Visible light and infrared light are more attractive to researchers. And combined with photothermal agent, the thermal stimulation response platform can also be constructed.

**FIGURE 5:**
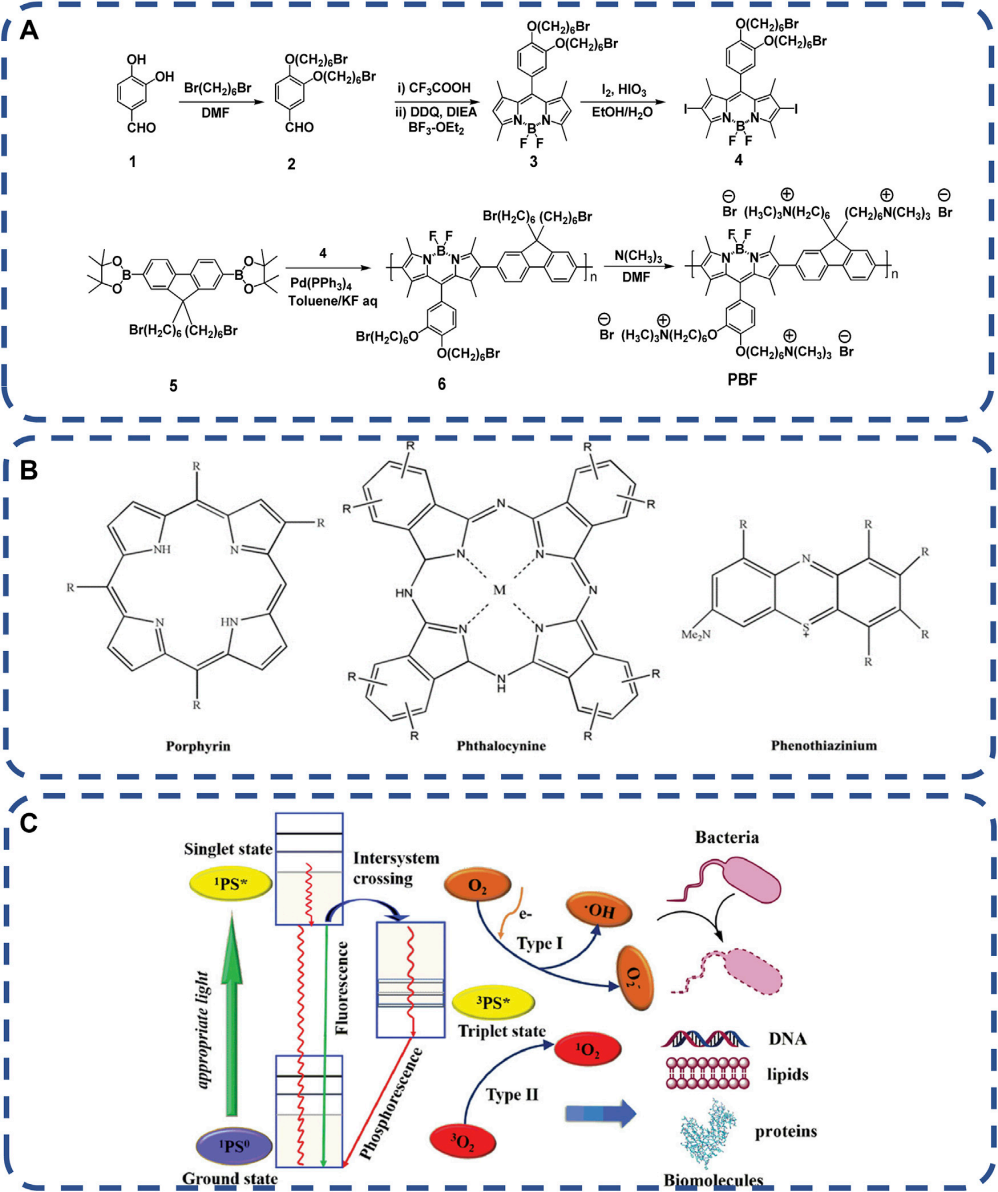
**(A)** Synthesis route of water-soluble polymer PBF (Gontsarik et al., 2019). Copyright 2012, Langmuir. **(B)** The basic chemical structure of classical PS used in PDAT, including porphyrins, phthalocyanines and phenothiazines, and the PDAT mechanism of **(C)** PSs under appropriate light emission (Jia et al., 2019). Copyright 2019, Adv Healthc Mater.

Among the available stimuli, non-invasive and essentially clean light is particularly attractive and suitable for biological systems without compromising normal function (Browne and Feringa, 2009; Szymanski et al., 2013; Borges et al., 2014; Qu et al., 2015). Photodynamic therapy (APDT) with non-invasive and spectral antimicrobial activity is a traditional technique. Active oxygen (ROS) produced by photosensitizer (PS) kills bacteria. Based on electrostatic interaction, positively charged ionic antimicrobial agents can efficiently destroy negatively charged bacterial cell membranes (Muñoz-Bonilla and Fernández-García, 2012; Xiong et al., 2014; Jiao et al., 2017). Azobenzene (Azo) is one of the commonly used light response molecules, which reversibly converts between the extended trans form and the dense cis form when exposed to ultraviolet and visible light (Jiao et al., 2017). Chong et al. (2012) synthesized fluorene and boron-dipyrromethene repeat units in the backbones (PBF). PBF can form uniform nanoparticles with disodium salt 3-dithiodipropionic acid (SDPA) through electrostatic interaction in aqueous solution, as shown in Figure 5A. Excited by 400 and 800 nm white light, PBF nanoparticles activate oxygen molecules and produce reactive oxygen species (ROS) to damage biomolecules, such as lipids, DNA and proteins, to quickly kill bacteria and cancer cells. In particular, PDAT does not require specific targeted interactions between photosensitizers (PSS) and bacteria, and bacteria are unlikely to develop drug resistance. The commonly used photosensitizers include macrocyclic compound-based PSs, non-self-quenching PSs, conjugated polymer-based PSs and nano-PSs (Jia et al., 2019), as shown in Figures 5B, C. Xing shuli et al. (Li et al., 2018b) demonstrated that a new phthalocyanine assembly, NanoPcA, has the ability to promote efficient ROS production through class I mechanisms.

Compared with the pure light response group, the photothermal response group is relatively more. APTT refers to photothermal agent (PTA), which converts light energy into heat energy to produce local hyperthermia to cleave the target bacteria (Ray et al., 2012; Pallavicini et al., 2014). The phenomenon of photothermal release is that electrons on the surface of conductive materials convert the light energy absorbed into thermal energy dissipation (Canaparo et al., 2019). Plasma active metal particles are very effective photothermal agents, such as silver nanoparticles, copper nanoparticles and gold nanoparticles. They have controllable optical properties, large light absorption cross section and high efficiency of converting light energy into thermal energy (Jaque et al., 2014). Graphene-based nanomaterials are typical PTA (Fan et al., 2018). Amoli Diva et al. embedded AgNPs into poly (butyl methacrylate-co-acrylamide-co-methacrylic acid) hydrogel to control the release of ofloxacin. The rats were irradiated with 405 nm laser for 15 s at 10, 30 and 50 min. After 70 min, the amount of ofloxacin released from these samples was significantly higher than that of unirradiated samples (Amoli-Diva et al., 2017). Photothermal conversion materials increase the Designability of photoresponsive nanoparticles and fabricate photoresponsive nano-platforms by simply combining photothermal agents and thermal response materials. Photothermal/antimicrobial therapy has great potential in eliminating bacterial infections (Moorcroft et al., 2020; Huang et al., 2022). Compared with other synthesis methods, photochemistry has several key advantages, including fine space-time control, and no need for heating or any solvent. Together with green organic chemistry, this field highlights the great potential of natural compounds (terpenes, polyphenols, polysaccharides, etc.) as cheap, renewable and safe basic materials (Versace et al., 2021). However, in some cases, the high temperature healthy tissue of APTT cannot be tolerated, resulting in healthy tissue damage. In addition, the efficacy of APDT is limited by local oxygen content, and the site of infection is always in anoxic environment. Beside of the combination of APTT or APDT with antimicrobials, the combination of both can also enhance the antibacterial efficacy (Wei et al., 2019).

#### 2.2.2 Electrical stimulation responsive material

**FIGURE 6:**
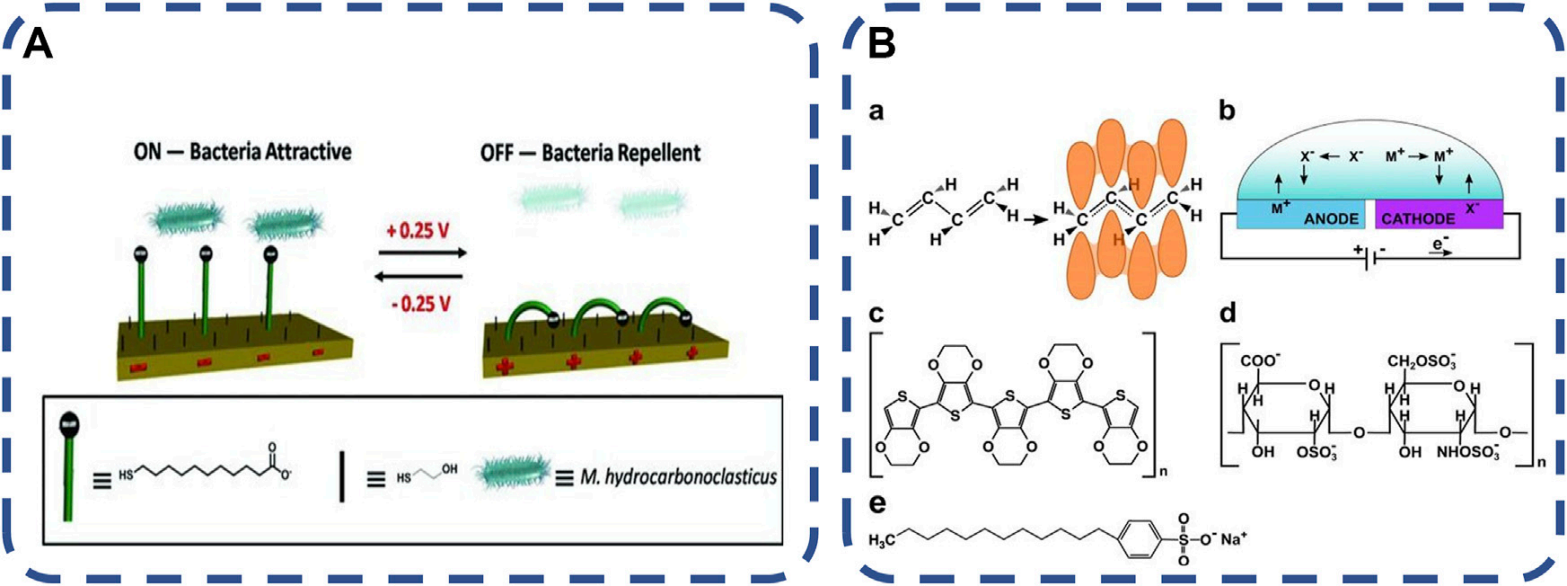
**(A)** Schematic diagram of an electrically switchable two-component SAM, which can switch its molecular conformation reversibly and rapidly according to the applied potential (Pranzetti et al., 2013). Copyright 2013, Adv Mater, **(B)** Chemical structure and electrochemical redox reaction in conductive polymer. a Chemical structure of the conductive polymer polyacetylene (left) and its resonance hybrid (right). b Ion flux in a conductive polymer-based electrochemical cell. m+ and x− represent positively and negatively charged ions, respectively. The blue color of the anode and the purple color of the cathode represent the electrochromic effect occurring in oxidized and reduced conducting polymers. c chemical structure of PEDOT. d chemical structure of heparin. (Gomez-Carretero et al., 2017). Copyright 2017, NPJ Biofilms Microbiomes.

In the field of skin and transdermal drug delivery, there have been a large number of literatures on the research of current used *in vivo*, which have determined the safety limit of applied electric field strength (Vanbever and Préat, 1999). Moreover, conductive polymers have been used to detect and regulate bacterial colonization, diagnose bacterial infection and prevent biofilm formation. Electric response delivery systems are often used to release drugs at specific locations and at specific times (Gomez-Carretero et al., 2017; Butina et al., 2019). The release location and drug dosage depend on the implantable polymer or electronic equipment using an external electric field. Compared with other methods, electrical stimulation has the advantages of rapid induction, high controllability of time and space, non-invasive, etc. (Pranzetti et al., 2013; Cantini et al., 2016). Bacteria are sensitive to electrical pulses, and electroactive materials containing specific compounds may allow bacterial biofilms to grow (Czerwinska-Glowka et al., 2021). One of the most studied polymers is poly (3mine4-ethylene dioxythiophene) (PEDOT), which has good environmental and electrochemical stability. When oxidized, PEDOT can promote bacterial adhesion and growth, as well as biofilm formation. This behavior may be caused by the existence of available sites for bacterial electron transfer. On the contrary, the reduced PEDOT film can express antibacterial activity. It is likely that the electron saturation on the surface of PEDOT prevents the electron transfer of bacteria (Pranzetti et al., 2013; Gomez-Carretero et al., 2017), as shown in Figures 6A, B. Because of its good biocompatibility, PEDOT is considered as an ideal candidate for various bioengineering applications. Dominika et al. (Czerwinska-Glowka et al., 2021) added antibiotics (tetracycline, Tc) to poly (3pyr4-ethylene dioxythiophene) (PEDOT) matrix to produce a new coating with obvious antibacterial activity against Gram-negative strains. The polymer can be used as a Tc carrier to participate in the design of powerful antibacterial systems with electrically triggered response, which provides a solid foundation for further medical applications, especially transplantation. The switching mechanism of electrical response to stimulation is based on the skeleton or end group of charged molecules, which has been proved to control the interaction between non-specific and specific biomolecules. Through the study of various dynamic molecular structures, it has become a powerful tool for regulating the interaction between the surface and proteins, bacteria and mammalian cells (Cantini et al., 2016). It is expected to open up new prospects in tissue engineering, drug delivery, biological imaging and regenerative medicine.

#### 2.2.3 Magnetic responsive material

The literature on the use of magnetic particles in drug delivery experiments first appeared in the 1960s. Freeman et al. (1960) proposed that magnetic nanoparticles can be transported through the blood system and concentrated in specific parts of the body through the external magnetic field. In the later development, some researchers used them in the research of tumors and developed new drug delivery systems containing drugs and magnetic nanoparticles (Hafeli et al., 1994). In recent years, researchers have combined this stimulus response system with infection treatment to develop a local drug delivery system for orthopaedic surgery or post-traumatic infection control (Harris et al., 2017).

**FIGURE 7:**
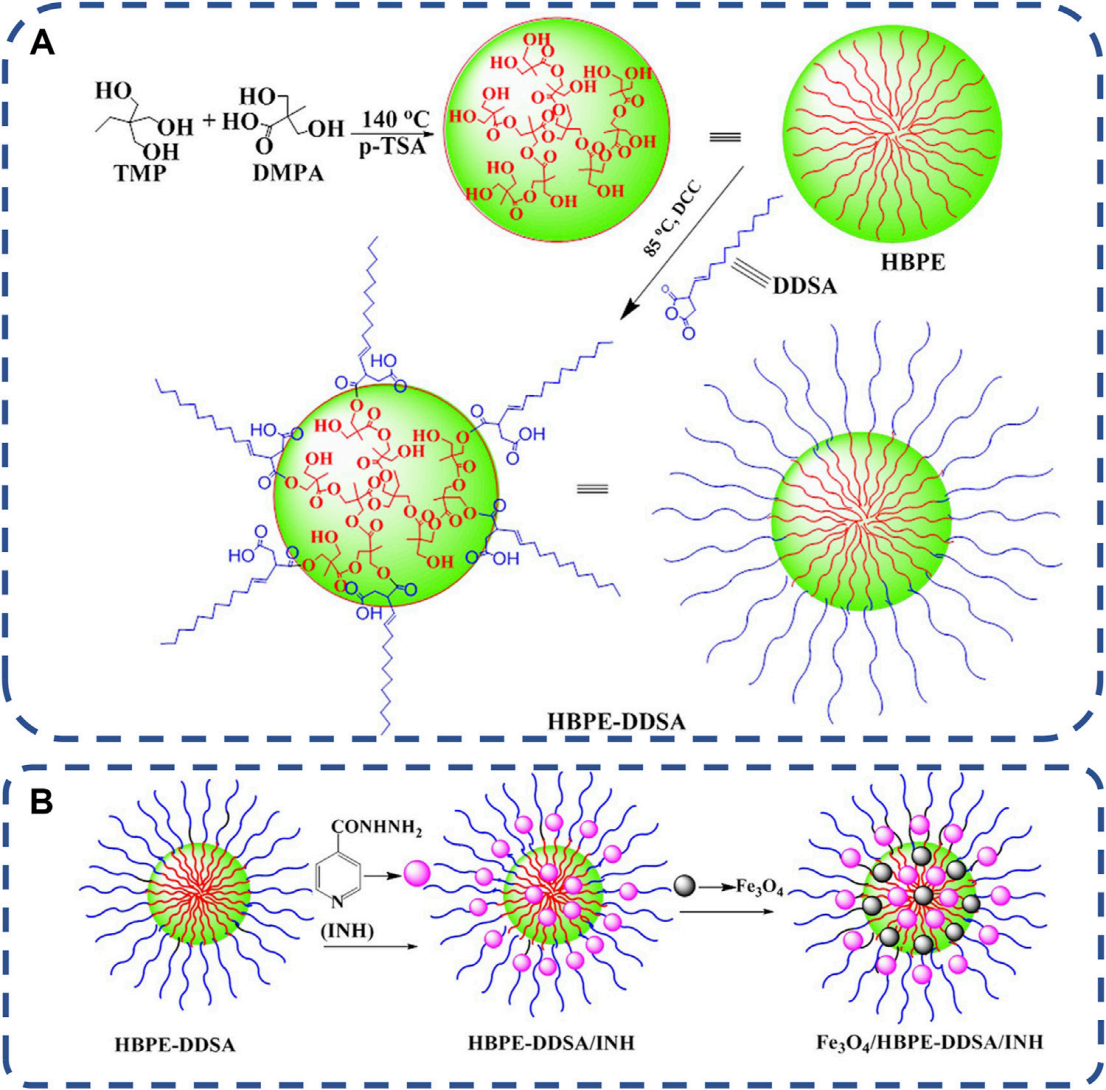
**(A)** Synthetic route of HBPE-DDSA. **(B)** The formation of the nanocomposites of HBPE-DDSA/INH and Fe3O4/HBPE-DDSA/INH (Zhao et al., 2017).Copyright 2017, J Biomater Sci Polym Ed.

In recent years, more and more attention has been paid to magnetic responsive drug delivery system. This is an efficient drug delivery system designed to target the delivery of drugs to specific organs or tissues of the body, improve therapeutic effectiveness and reduce or eliminate adverse drug side effects (Dobson, 2006; El-Husseiny et al., 2011). It is easy to use and easy to target to deeper tissue carriers (Kumar and Mohammad, 2011). The most commonly used superparamagnetic nanoparticles (MNP) in magnetic responsive delivery systems are Fe_3_O_4_, which can be magnetized under the influence of an external magnetic field for *in vivo* applications. However, the exposed Fe_3_O_4_ nanoparticles have poor stability and are easy to agglomerate. Therefore, it is a correct choice to choose the nanocomposite carrier with good biodegradability, good mechanical properties and biocompatibility as the matrix for Fe_3_O_4_ incorporation (Zhao et al., 2017). Hyperbranched polyester HBPE can provide structural support and functionalize Fe_3_O_4_, thereby improving the dispersion and stability of Fe_3_O_4_ particles, as shown in Figures 7A, B. Chi lizhao et al. (Zhao et al., 2017) synthesized HBPE-DDSA polymer from dodecenyl succinic anhydride (DDSA) functional group and hyperbranched polyester (HBPE). Then superparamagnetic iron oxide Fe_3_O_4_ nanoparticles were dispersed in HBPE-DDSA to synthesize magnetic nanocomposites Fe_3_O_4_/HBPE-DDSA. Finally, Fe_3_O_4_/HBPE-DDSA was combined with isoniazid. The experimental results show that the nanocomposites show good superparamagnetic behavior, non-toxic and good biocompatibility, and have great potential as targeted drug delivery carriers.

Chitosan is also a magnetic responsive drug carrier. Paramagnetic Fe_3_O_4_ nanoparticles and antibiotic vancomycin were encapsulated into chitosan beads and crosslinked with polyethylene glycol dimethacrylate of different lengths to obtain magnetic responsive polymers (Harris et al., 2017). 30 min of magnetic stimulation increased the daily drug elution rate of the polymer. Magnetic stimulation can be used to increase drug delivery after implantation to obtain maximum drug concentration or to maintain therapeutic drug levels after controlling the elution rate required by traditional delivery systems, it can be used as a potential infection prevention and treatment device.

### 2.3 Multiple stimulus responsive antibacterial materials

The multi-stimulus response antibacterial system can detect bacteria in time, respond to bacterial metabolites and release drugs according to the infection site, which improves the germicidal efficacy and biocompatibility (Li et al., 2018c). Many double stimulus or multiple stimulus response materials have been developed. This multiple stimulus response antibacterial material has the advantages of drug release and enhancing therapeutic efficacy. Lot’s of stimulus-responsive materials showed surface renewable properties through the changes of response light, pH value and temperature because of their interfacial properties (Cole et al., 2007; Zhao et al., 2012).

The dual response of thermal response and pH response can effectively enhance the antibacterial effect of the material and inhibit the attachment of bacteria (Elashnikov et al., 2022). Stimulation-responsive antibacterial nanofibers were prepared by electrospinning poly (caprolactone) (PCL), poly (N-isopropylacrylamide-co-acrylamide) PNIPAm-co-AAm with different concentrations and ciprofloxacin (CIP). The low critical solution temperature (LCST) of PNIPAm-co-AAm was determined by refractometry in distilled water and buffer solution of pH4 and 7.4. The nanofibers showed enhanced release at temperatures lower than LCST. Compared with PCL nanofibers loaded only with CIP, the adhesion of two kinds of bacteria on PCL/PNIPAM-Co-AAM containing CIP decreased significantly. Ni et al. (2021) designed triple functional smart surfaces by topologically integrating temperature-responsive poly (N-isopropylacrylamide) NIPAM, photoresponsive dissociation of azobenzene/cyclodextrin (Azo/CD) complexes, hydrophilic PHEMA fragments and nano-fungicides (AgNPs) on a single substrate. Due to the thermosensitive conformational changes of PNIPAM fragments and the synergy of host-guest interactions between Azo and CD derivatives, it can further release more than 94.9% of previously killed bacteria.

pH and Redox response Materials. The pH values of different organs/tissues were significantly different in the state of disease, and the pH value of extracellular fluid was 7.35–7.45 normally. The pH of different organelles is also slightly different, Golgi 6.4, endosome 5.0–6.0 and lysosome 4.0–5.0 (De Arco et al., 2009). The difference in pH between normal and diseased tissues has been used as a response to stimuli that trigger drug release (Ganta et al., 2008; Pan et al., 2012). Curcio et al. (2017) prepared pH/redox double-response nanogels (DEX-SS) using methacrylate dextran (DEXMA) and 2-aminoethyl methacrylate (AEMA) as the response part of pH/and N-mathyl-bis (acryloyl) cystamine (BAC) as redox response cross-linking agent. Then loaded with methotrexate (MTX), pH/redox double-response nanogels (DEX-SS) were obtained. GSH released methotrexate (MTX) in the environment of pH 5.0 and 7.4, respectively, which proved the pH and redox response of DEX-SS nanogels. The results showed that the release rate of methotrexate increased by 5 times in acidic environment.

PH and high reactive oxygen species (ROS) dual response materials. Ye et al. (2021) reported a dextran-coated stimulus-responsive nanoparticles (NP) encapsulated with a hydrophobic antibiotic rifampicin. NP showed strong affinity for a variety of pathogens *in vitro* and could effectively accumulate in bacterial infected tissues. NP is activated by low pH and high reactive oxygen species in the infective microenvironment, releasing cationic polymers and rifampicin, which have synergistic activity against AMR pathogens. Poly (β-aminoester)-guanidine-phenylboric acid (PBAE-G-B) polymer with disulfide bond in its main chain is easy to degrade into non-toxic by-products, which further enhances its biocompatibility (Hwang et al., 2011). NP can effectively eliminate biofilm and intracellular infections and resist AMR pathogens *in vitro* and *in vivo*. Shi et al. (2021) developed a novel dynamic hydrogel based on dynamic covalent bond of borate esters using phenylboric acid modified hyaluronic acid (HA-PBA) and polyphenol-tannic acid (TA). Dynamic hydrogels can be used for pH responsive and reactive oxygen species (ROS) responsive release of antibiotics without obvious cytotoxicity and hemolysis and good histocompatibility.

Double response of electricity and pH. The drug release of conductive nanoparticles can be controlled by applying a weak external DC electric field. This method represents a new responsive drug delivery system that effectively controls the time, space and dose of drug release. A drug delivery system based on temperature and electric field stimulation. Qu et al. (2018) proposed an electric field and pH stimulus response system and developed injectable conductive hydrogels with electrical responsiveness, pH sensitivity and inherent antibacterial activity as drug carriers. Chitosan-graft-Polyaniline (CP) copolymer and oxidized dextran (OD) were mixed as crosslinking agents to prepare hydrogels. When the applied voltage increased the release rate of the model drug loaded in CP/OD hydrogel increased significantly. Both chitosan and Polyaniline have inherent antibacterial properties, which make hydrogels have excellent antibacterial properties.

PH/glucose double response. Liang et al. (2022) developed a class of antibacterial hydrogel dressings with good antioxidant capacity, appropriate mechanical properties, good hemostasis and conductivity, and pH/glucose double reactive drug release ability. It is used to repair the wound of exercise-induced diabetic foot. The polymer is based on the double dynamic bond of Schiff base and phenylborate bond. The structure of Schiff base is easy to dissociate under acidic conditions. The competitive binding of phenylboric acid with glucose leads to the dissociation of the coordination structure of catechol and phenylboric acid. This double dynamic bond makes the gel have double reactivity between pH and glucose, which is beneficial to the release of therapeutic drugs (Shan et al., 2017; Shi et al., 2020).

### 2.4 Antibacterial substances

Bacterial extracellular matrix (ECM) is mainly composed of proteins, extracellular polysaccharides and eDNA polymers (EPS) matrix structure. Provide a barrier for biofilm bacteria to resist the killing of antibiotics, or pump antibiotics through an efflux pump (Limoli et al., 2015). In recent years, for the control of antibiotic drugs, the development of bacterial drug resistance has slowed down, but there is urgent to develop the next-generation of spectral antibiotics or alternative therapy. In this section, we describe the current situation and development prospects of antibiotics.

#### 2.4.1 Antibiotic

Antibiotics have been a popular treatment against bacterial infection in clinic in recent decades, saving countless infected patients. The main categories of antibiotics are β- Lactamides, glycopeptides, macrolides, oxazolidones, amphetamines, lincomamides, fluoroquinolones, nitroimidazoles, lipopeptides and polymyxin, etc. (Singh et al., 2017). β- Lactam antibiotics inhibit cell wall synthesis by combining with a series of enzymes and bacteria, thereby inhibiting bacterial growth. Vancomycin, the representative drug of glycopeptide antibiotics, can bind with d-alanine-d-alanine, the terminal dipeptide of Lipid II, the precursor of bacterial cell wall peptidoglycan chain, thereby preventing cell wall synthesis (Dougherty and Pucci, 2012). Lipopeptides such as daltomycin can be inserted into the cell membrane, leading to cell depolarization and the formation of cell membrane pores, ion leakage and the destruction/rupture of the cell membrane, leading to bacterial death. Aminoglycoside and tetracycline antibiotics bind to 16S rRNA of 30S ribosomal subunit to inhibit bacterial protein synthesis (Armstrong et al., 2012). Macrolide antibiotics bind to 23S rRNA of bacterial 50S ribosomal subunits to inhibit bacterial protein synthesis (Chellat et al., 2016). Oxazolidinone is a kind of synthetic antibacterial agent represented by linezolid, which also binds to 23S rRNA of 50S ribosomal subunit of bacteria to inhibit protein synthesis (Chellat et al., 2016). Quinolones inhibit bacterial growth by inhibiting bacterial DNA helicase, DNA synthetase and topoisomerase IV (Jacoby and Hooper, 2012). For orthopedic infectious diseases, no matter whether surgical treatment is performed or not, antibiotics are indispensable weapons in the hands of surgeons. Especially for patients infected with drug-resistant bacteria, scientific drug programs should be used to quickly kill possible pathogens and prevent infection complications. For patients infected with drug-resistant bacteria, the following medication principles are generally adopted: 1) When there is an infection beyond the control of an antibiotic and a mixed infection caused by multiple bacteria, combined medication is often required, such as β-lactam antibiotics (penicillin and ceftazidime) inhibit the synthesis of bacterial cell wall, belonging to the bactericide of reproductive period; Aminoglycoside antibiotics (tobramycin and gentamicin) inhibit bacterial protein synthesis, which belongs to the stationary phase bactericide. The combination of the two can strengthen each other; 2) The dosage of drugs should be controlled within the range that can form effective concentrations in blood and tissues without toxic and side effects. For example, glycosides and quinolones have concentration dependent bactericidal effects, that is, increasing the concentration can enhance the effect. Therefore, taking instantaneous high concentration pulse administration can not only enhance the efficacy, but also reduce the toxic and side effects; 3) For non-dose dependent drugs, such as β- Lactam antibiotics have different characteristics of action. Ultra-high concentration cannot strengthen its bactericidal effect, while maintaining the effective concentration in blood and tissue for a long time can improve the efficacy. Continuous release of drugs can enhance the efficacy. However, the combination of β-lactam antibiotics and aminoglycoside antibiotics has the risk of antagonism. The mechanism is that the amino groups of aminoglycoside antibiotics β-Amides with no biological activity are formed between the lactam rings, which will reduce each other’s efficacy. How to accurately manage the drug delivery mode is particularly important. However, the drug resistance of bacteria has also been increasing in recent years. Some studies have suggested that bacterial drug resistance exists long before clinical use (Bhullar et al., 2012; Waglechner et al., 2021), but bacteria that abuse antibiotics in clinical and agricultural fields that lead to antibiotic resistance (AMR) or multiple drug resistance (MDR) have been screened out. At present, pharmaceutical companies and experimental centers are carrying out the development of antibiotics (Hutchings et al., 2019), and 45 drugs are being tested, among which there are also categories of new modes of action. In the future, it is necessary to use computer simulation calculation, which is similar to the research of epidemic diseases (Chen et al., 2021; Wieland and Mercorelli, 2021). For example, through the computer simulation of targeting combined with bacterial modeling, *in vitro* activity of antibiotic molecules, safety screening, *in vitro* antibacterial activity of clinical strains and other screening processes, the best bactericidal mechanism will be finally determined, and a series of antibiotics suitable for orthopedic treatment will be designed to form a new antibiotic library.

#### 2.4.2 Metal nanoparticles

With the rapid development of nanotechnology, metal nanoparticles (NPs) have shown excellent properties in the field of antibacterial and conducive to medical applications, including excellent photoelectrochemical activity, large surface area-volume ratio and strong particle surface activity (Jaque et al., 2014; Pallavicini et al., 2014; Canaparo et al., 2019). Metal nanoparticles can kill microorganisms through a variety of mechanisms, so drug resistance is unlikely (Fan et al., 2018). Metal nanoparticles are mainly silver (Ag), zinc (Zn), copper (Cu), magnesium (Mg) and titanium (Ti). Metal nanoparticles containing alumina particles (Al2O3NP) are an exception and may promote the development of drug resistance (Huang et al., 2022).

**FIGURE 8:**
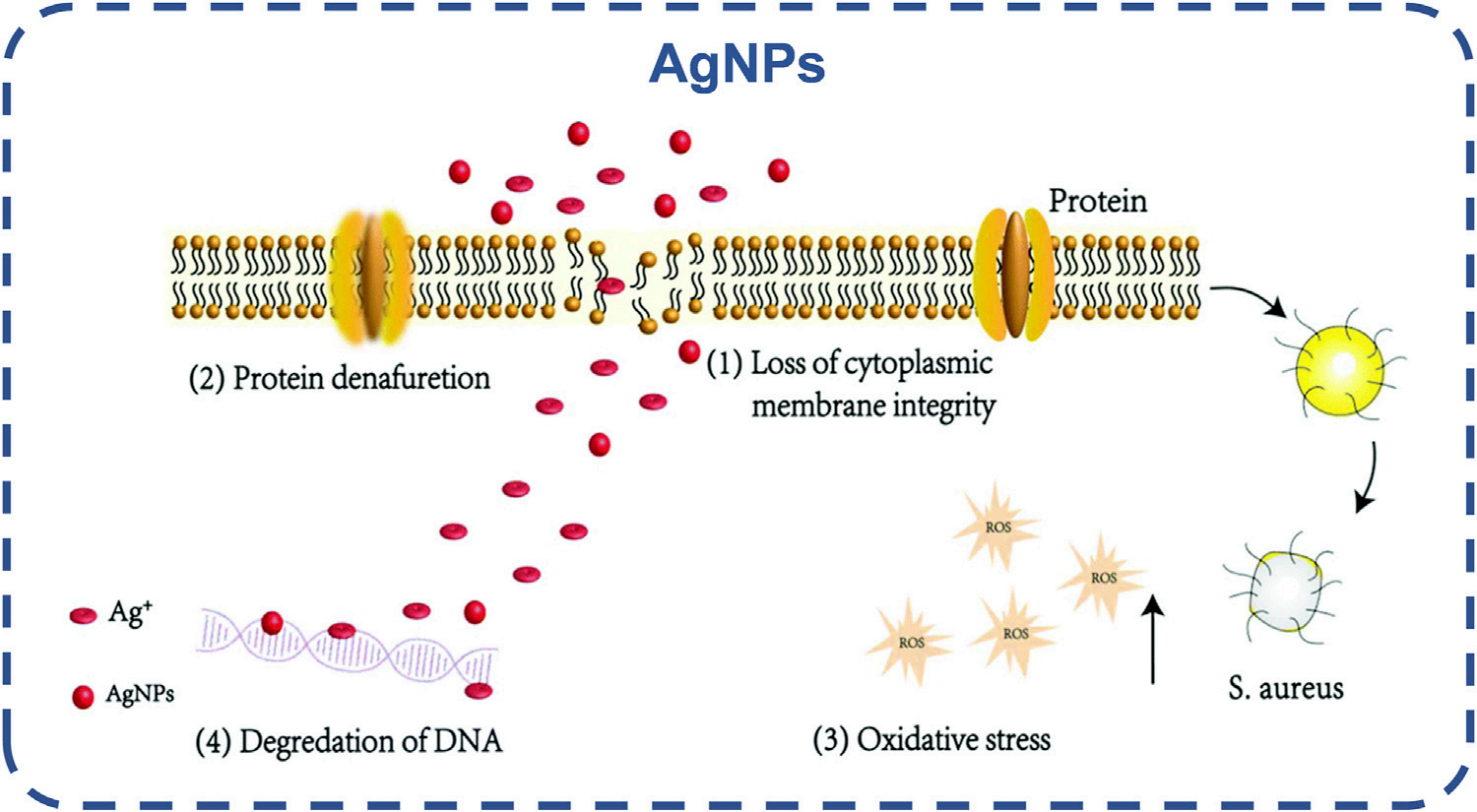
Schematic diagram of the potential antibacterial mechanism of AgNPs and Ag (Ding et al., 2020). Copyright 2020, Biomater Sci.

Silver nanoparticles (AgNPs) are the most common. AgNPs can destroy the bacterial cell wall, dissociate its peptidoglycan structure from inactivation, disrupt bacterial permeability, but also destroy respiratory proteases, disrupt the respiratory chain, leading to bacterial death biofilm disintegration (Morones-Ramirez et al., 2013; Jia et al., 2016; Ding et al., 2020), as shown in Figure 8. By inactivating bacterial proteins, AgNPs can penetrate bacteria, dephosphorylate tyrosine residues, and inactivate bacterial growth-related enzymes to inhibit bacterial growth (Cozzone, 2005; Welburn et al., 2007). Ag nanoparticles hinder the transfer of electrons, and oxygen is forced to become an electron acceptor, leading to the production of reactive oxygen species (ROS), such as hydroxyl radicals and superoxide radicals. Affecting DNA replication, Ag can transform DNA into a condensed state, thus organizing DNA replication (Marambio-Jones and Hoek, 2010; Xu et al., 2021a). There have been studies on the combination of stimulus response platform and AgNPs to develop a silver nano platform with stimulus response and controlled release (Guo et al., 2019). In the future, it is possible to achieve the controlled release of Ag ion and selectively kill bacteria, and alleviate the biological toxicity caused by the high concentration of Ag ion.

Zinc oxide nanoparticles (ZnONPs) also have a variety of mechanisms for killing bacteria, such as destroying lipids and proteins, resulting in the formation of Zn^2+^ ions and reactive oxygen species (ROS) to damage cells (Blecher et al., 2011; Huh and Kwon, 2011; Hajipour et al., 2012). Other metal ions have similar mechanisms for killing bacteria, but they are different. In general, it is difficult for bacteria to produce resistance (Pelgrift and Friedman, 2013). However, attention should be paid to the side effects of some metal ions, such as the toxicity of AgNPs and the skin hypersensitivity caused by TiO_2_.

#### 2.4.3 Antibacterial peptides (AMP)

Most eukaryotic biosynthetic AMP can target the plasma membrane of bacteria, dissolve the plasma membrane and destroy the bacterial structure (Ageitos et al., 2017; Torres et al., 2019). The main bactericidal mechanisms are membrane lytic activity, active oxygen (ROS) induction, enzyme inactivation, etc (Andoy et al., 2020). Antimicrobial peptides can also destroy the bacterial biofilm through three aspects: cleavage of eDNA (Whitchurch et al., 2002), destruction of bacterial quorum sensing (QS) (Herget et al., 2017; Whiteley et al., 2017) and destruction of the whole biofilm (Chen et al., 2018). According to its source, AMP can be divided into two types (Xu et al., 2021b): one is natural antibacterial enzyme, which is called host defense peptide (HDP) or defensin. Because of its targeted antibacterial mechanism, HDP has spectral antibacterial activity, but it is easy to be hydrolyzed, poor stability, moderate activity and expensive. The other is the synthetic peptide (HDPs) that mimics HDP artificially, which is the modification or improvement of HDP, has higher stability, resistance to protein hydrolysis and cheap. Peptides synthesized by bacterial ribosomes, called bacteriocins, should never be removed by bacteria. Compared with antibiotics, cationic amphiphilic bacteriocin can also penetrate phospholipase when adhering to bacteria. Bacteriocin exerts its antibacterial effect by inhibiting cell wall synthesis, inhibiting cell membrane formation and interfering with DNA and protein synthesis at high concentrations (Meade et al., 2020). Antimicrobial peptides can also have self-assembly properties. Liu and his colleagues (Liu et al., 2013) selected a Gram-positive antimicrobial peptide as the basic framework and developed a self-assembly material. When exposed to pH, ion or thermal stimulation, the sequence containing antimicrobial peptides can make a reversible transition from random structure to β-fold structure, and further self-assemble into hydrogels with antibacterial function on their surface. Similar to antibiotics, bacteria can evolve resistance to AMP, which will become more and more obvious in the future (Andersson et al., 2016). Because of their specific binding, some antimicrobial peptides show high selectivity for anticancer and killing cancer cells, and are harmless to normal cells. Vectors constructed with functional polymers can help kill cancer cells that escape from immune system monitoring (Sun et al., 2018).

## 3 Conclusion and prospect

In general, all material systems have their own characteristics, but the general policy is to precisely control the release of drugs. For various substances, their modes of action, ways and possible application prospects are different. The enzyme stimulating response material system can react with certain specific enzymes produced by bacteria, such as glutamyl endonuclease, lipase, β- The lactamase, combined with antibacterial substances such as Ag ions, has an adaptive drug release function to play a good antibacterial role. It has good application prospects in biological materials, tissue engineering and regenerative medicine, such as integrating the response system with the orthopedic implant system to prevent infection. The advantages of the salt responsive material system can be switched between the antibacterial surface that kills and resists living bacteria and the antifouling release surface that releases dead bacteria. It can be easily regenerated through simple salt solution treatment to maintain its high antibacterial and antifouling activities, thus playing an antibacterial role, having good biocompatibility and high bacterial clearance rate. It is expected to provide a new idea for the design of orthopedic built-in functional materials with ideal antibacterial surfaces. The advantage of pH responsive material system is that it is sensitive to pH changes and will not release drugs under physiological pH. For acidic inflamed environment, it can rapidly increase the local concentration of antibacterial drugs and has a strong antibacterial effect. For example, based on the acidic environment in osteomyelitis, this material system has the potential of bone substitute and can also be used as the stimulus response nano carrier of macro drug delivery system. For different design schemes, there are different uses. For the application of redox response material system, due to different metabolites of bacteria, this material can be used to distinguish Gram negative and Gram-positive bacteria, identify different reduction products secreted by different bacterial strains, and select and specifically detect or kill bacteria. The photosensitizer of light stimulated antibacterial therapy does not need the antibacterial method of bacterial target, and is not easy to produce drug resistance. It is suitable for local infection. The research of new photosensitizer and optical technology can improve its antibacterial depth. The electric trigger response system has many characteristics and fast response speed. Using the low drive voltage and magnetic field compatible with the biological system, multiple switchable regions can be created on the same surface. Applying potential to change the surface characteristics will change the effect of the antibacterial surface on bacteria, thus affecting their viability. Combining antibacterial drugs with them can form materials with adjustable antibacterial properties. In active stimulation, the magnetic stimulation system is easy to use and targets deep tissues. Magnetic stimulation can be used to increase the drug delivery after implantation to obtain the maximum drug concentration, or maintain the therapeutic drug level after the traditional delivery system is lower than the required release rate. In this paper, the research status of stimulus response system and the development of antibacterial substances and potential applications in bone infection were reviewed. Bacterial biofilm and drug resistance are the main problems in dealing with bacterial infection, and the resistance of bacteria to antibiotics is becoming more and more intense. Although many countries and organizations have regulated and restricted the use of antibiotics, they only slow down the growth trend. Stimulus-response antibacterial system has developed rapidly in recent years, and various components and metabolites of bacteria have been studied. The endogenous stimulus response system includes pH response, salt response, enzyme response, etc. The response systems of exogenous stimuli include light response, thermal response, magnetic response, electrical response, etc. Each system has its own applicable environment. When the tissue has pathological changes, the changes are in many aspects, and the pH value and metabolites are constantly changing. Therefore, when a defect in the single response system, it can be combined with other systems to form a multi-stimulus response system, which is conducive to the better role of therapeutic drugs. At present, with the increasing resistance to antibiotics, the research and development of antibacterial substances is accelerating. There are not only the improvement of traditional antibiotics and the optimization of antibiotic discovery pathway. With the development of nanotechnology, a variety of fungicides began to enter people’s field of vision, such as metal nanoparticles, which have advantages in sterilization. However, the development of its technology is not enough to be used in bone clinic. The development prospect of stimulus response system and antibacterial materials has partial unity, towards highly sensitive materials, precise control of drug release, and selective action on bacteria. This method has a good prospect, but it requires considerable energy and effort, and at the same time, it is necessary to make these systems and antibacterial materials as simple and efficient as possible.
